# The characteristics of dyslipidemia patients with different durations in Beijing: a cross-sectional study

**DOI:** 10.1186/1476-511X-9-115

**Published:** 2010-10-13

**Authors:** Yingying Liu, Puhong Zhang, Wei Wang, Huan Wang, Ling Zhang, Wei Wu, Xiuhua Guo

**Affiliations:** 1 Department of Epidemiology and Health Statistics, School of Public Health and Family Medicine, Capital Medical University, No.10 Xitoutiao, You An Men Wai, Beijing, China; 2 Xuanwu District Centre for Disease Control and Prevention, No. 34, Changchunjie Xuanwu District, Beijing, China; 3 Institute of Chronic and Noncommunicable Disease Control and Prevention, Beijing Center for Disease Prevention and Control, Beijing, China

## Abstract

**Background:**

Prevalence of dyslipidemia is high and increases even in younger people. The key aim of this study was to explore the group characteristics of patients in different durations of dyslipidemia and provide clues for the management of dyslipidemia in Beijing.

**Results:**

Patients with short duration of dyslipidemia were mainly characterized by relatively young age, occupational groups, not eating or irregular eating breakfast, less physical activities, having the habit of smoking, and 53.8% is with abnormal LDL-c, 10.4% is with abnormal HDL-c, and 51.5% is with abnormal TG. 54.6% of patients with longer duration is with abnormal LDL-c, 12.8% of them is with abnormal HDL-c, and 57.1% is with abnormal TG. They paid much more attentions to their health, tried to eat breakfast regularly and do more physical activities, gave up smoking, and had regular breakfast, but increasing physiological disorders such as elevated blood pressure and glucose appeared. Severe sequelaes (stroke, myocardial infarction) were mainly observed in patients with the duration of more than 10 years. And in this group the proportions of patients with LDL-c ≥ 4.15 mmol/L and TG ≥ 4.53 mmol/L are the highest among the three groups.

**Conclusions:**

we should strengthen the tertiary prevention and improve the control rate of dyslipidemia in Beijing. Health promotion programs such as tobacco control and physical exercise should be carried out for younger patients.

## Background

Dyslipidemia, a common lipid abnormality is characterized by elevated lowdensity lipoprotein cholesterol (LDL-c), elevated triglycerides (TGs), or low high density lipoprotein cholesterol (HDL-c) [1]. Prevalence of dyslipidemia is high and becomes to increase even in younger people[2]. In addition to elevated LDL-c, both low HDL-c and elevated TG are increasingly being recognized as independent risk factors for coronary heart disease (CHD)[3,4]. Dyslipidemia is one of the leading causes of death and cardiovascular morbidity in western countries[5]. Hypertension, dyslipidemia, endothelial dysfunction and oxidative stress are the major pathologies involved in CVDs and impose a great risk[6]. Dyslipidemia is responsible for 54% of population attributable risk for myocardial infarction (MI)[7,8]. Dyslipidemia is also an important contributor to cardiovascular risk in people with metabolic syndrome[6]. Dyslipidemia is consanguineously related with life style[9]. Better high-density lipoprotein (HDL) can be gotten through changing the lifestyle[10]. And hypercholesterolaemia is the permissive factor that allows other risk factors to operate[11]. If the TC decreased 1%, the incidence of CHD will reduce 2%. And if TC decreased 10%, the mortality of CHD will reduce 13%~14%[12].

The importance of dyslipidemia management is based on cardiovascular risk factors. Assessment of the patient's risk for coronary heart disease helps determine which treatment should be initiated[13]. The lipid management goal is also based on risk assessment and the management of dyslipidemia doesn't always require drug therapy. Particularly, lifestyle modification is important for the management of low HDL-C and TG[14]. We manage to explore the group characteristics of patients in different durations of dyslipidemia through a cross-sectional investigation which will assess cardiovascular risk factors of the dyslipidemia patients and provide clues for the prevention and therapy of dyslipidemia in Beijing.

## Methods

### Survey methodology

A cross-sectional study was performed to research the prevalence level of chronic diseases and risk factors in Beijing in 2005, in which, the target people involved was aged ≥ 18 years old and had been living in Beijing for at least 6 months. In the study, 19216 individuals who represented 19216 families involved in the survey with multi-stage cluster sampling study, and this investigation covered 162 communities of 54 sub districts in all 18 municipal districts. The sub districts were sampled with probability proportional to size cluster sampling method in each district. 16711(87.0%) subjects were valid and used in consequent studies. And 2692 diagnosed dyslipidemia patients with complete duration information from the 16711 valid participants were chosen for the correspondence analysis in this study.

Our survey included a questionnaire, physical measurements, blood pressure measurements and laboratory tests to collect information. Before the investigation all the people involved had signed the informed consents. We used Calender 7600 autoanalyzer to test fasting lipid components for the diagnosis of dyslipidemia in the laboratory of Beijing CDC(the Center for Disease Control and Prevention).

### Diagnosis methods and a layered approach

Dyslipidemia was diagnosed as any abnormal status of LDL-C, HDL-C and TG (based on the standard of ATPIII: TG ≥ 1.70 mmol/L, LDL-c ≥ 3.46 mmol/L, HDL-c < 0.91 mmol/L), And basing on the standard of ATPIII, TG was divided into four groups(TG < 1.70 mmol/L, 1.70 ≤ TG < 2.27 mmol/L, 2.27 ≤ TG < 4.52 mmol/L, TG ≥ 4.53 mmol/L); LDL-c was divided into three groups(LDL-c < 3.46 mmol/L, 3.46 ≤ LDL-c < 4.15 mmol/L, LDL-c ≥ 4.15 mmol/L); HDL-c was divided into three groups(HDL-c < 0.91 mmol/L, 0.91 ≤ HDL-c < 1.56 mmol/L, HDL-c ≥ 1.56 mmol/L). The duration of dyslipidemia was calculated according to the date on which one was first diagnosed as dyslipidemia patient. Body mass index (BMI) was calculated as body weight divided by height squared (kg/m^2^). Lower-weight(L-W), normal-weight(N-W), overweight and obesity were defined as BMI < 18.5, 18.5 ≤ BMI < 25, 25 ≤ BMI < 30 and BMI ≥ 30, respectively.

Current smokers included regular and casual smokers within the last month and were classified as non-smoking, 0-9 cigarettes/day, 10-19 cigarettes/day, 20-29 cigarettes/day and ≥ 30 cigarettes/day. Drinking groups included non-drinking (never drink or drink less than 1 time per month), occasional drinking (drink more than 1 time per month but less than 2 times per week) and frequent drinking (drink more than 2 times per week). Lack of physical activities referred to less than 2 hour physical activities per week including walking, dancing, running, swimming and court game, excluding work purpose physical activities. Low-intake of calcium, low-intake of fruit and vegetable and protein intake were all based on food frequency questionnaire.

According to the measurement of blood pressure(BP), we defined the normal BP, borderline hypertension, low-grade hypertension, middle-grade hypertension and high-grade hypertension as SBP < 130 mmHg and DBP < 85 mmHg, 130 mmHg ≤ SBP < 140 mmHg or 85 mmHg ≤ DBP < 90 mmHg, 140 mmHg ≤ SBP < 160 mmHg or 90 mmHg ≤ DBP < 100 mmHg, 160 mmHg ≤ SBP < 180 mmHg or 100 mmHg ≤ DBP < 110 mmHg, SBP ≥ 180 mmHg or DBP ≥ 110 mmHg. And according to the fasting plasma glucose (FPG), normal FPG, impaired fasting glucose(IFG) and diabetes were defined as FPG < 6.1 mmol/L, 6.1 mmol/L ≤ FPG < 7.0 mmol/L and FPG ≥ 7.0 mmol/L. We diagnosed metabolic syndrome (MS) as the standard of ATPIII. The consideration of myocardial infraction and stroke were according to the diagnosis of local hospitals.

The sample size of each duration group was: "0-4 years": 1816 (67.5%); "5-9 years": 517 (19.2%); " ≥ 10 years":359 (13.3%).

### Statistical analysis

All data were doubly input and checked with Epidata3.1 by a professional data recording company. Abnormal values and missing values were checked (logic check) by quality control group to ensure the data accuracy. SAS software (version 9.1, SAS Institute (Shanghai) Co., Ltd.) was used to perform univariate analysis and multiple correspondence analysis. Prior to multiple correspondence analysis, univariate analysis were made to find significant variables. Nonparametric test was used to explore the correlation between a particular correlated factors and durations of dyslipidemia. We performed Mann-Whitney with the variables classified as two levels, and Kruskal-Wallis for variables classified as multilevel out of orders (such as occupation), and Spearman rank correlation analysis for variables classified as multilevel orders.

Correspondence analysis is used to analyze the differences among every sort of one same variable and the corresponding relationship among every sort of variable. This method is converting an original data matrix X = (x) nm contains n subjects and m variables into another matrix Z = (z) nm, and also making R = Z'Z (covariance matrix analyzing the relationship among variables) and Q = ZZ' (covariance matrix analyzing the relationship among subjects) have the same non-zero eigenvalue by utilizing a sort of data transformation method. The horizontal axis of the correspondence analysis graph is the first dimensionality, and the second dimensionality is the vertical axis. The distance between the two variables can indicate the approximate relationship between the two variables[15,16].

## Results

### The awareness and control rates of dyslipidemia are low in Beijing

According to the new test results of TC, TG, LDL, HDL, there are 6709 dyslipidemia patients among 16711 subjects. And the prevalence rate of dyslipidemia is 40.15%. And according to the questionnaire, the awareness, control rates of dyslipidemia were 43.79% and 17.20% respectively. 17.35% of the patients control dyslipidemia through taking drugs, 3.61% of the patients through dietary restriction and 10.51% through physical exercises. Among 6709 dyslipidemia patients, 2692 cases can recall when and where their dyslipidermia were diagnosed while 249 patients can't. And 3768 patients are newly diagnosed through our investigation. Among the 2692 patients with complete duration information, 7.0%(188) was diagnosed in Community Health Station, 13.2%(354) was diagnosed in Health service centers in Communities, 48.0%(1293) was diagnosed in Chinese Level II Hospital, and 31.8%(853) was diagnosed in Tertiary Health Care. And the lipid degrees and BMI of dyslipidemia patients with different durations is shown in table 1.

**Table 1:** the lipid level and BMI of dyslipidemia patients with different durations

		0~4 years		5~9 years		10 years~		Statistic	P value
						
		n	%	n	%	n	%		
LDL-c (mmol/L)	<3.46	839	46.2	235	45.4	154	43.1		
	3.46~	544	30.0	141	27.3	95	26.5		
	≥ 4.15	433	23.8	141	27.3	109	30.4	0.037	0.057
HDL-c (mmol/L)	<0.91	189	10.4	66	12.8	45	12.6		
	0.91~	1365	75.2	378	73.1	260	72.6		
	1.56~	262	14.4	73	14.1	53	14.8	-0.020	0.308
TG (mmol/L)	<1.70	881	48.5	222	42.9	166	46.4		
	1.70~	352	19.4	105	20.3	63	17.6		
	2.27~	457	25.2	144	27.9	94	26.3		
	≥ 4.53	126	6.9	46	8.9	35	9.8	0.045	0.020
BMI	L-W	12	0.7	1	0.2	1	0.3		
	N-W	449	24.7	118	22.8	83	23.2		
	overweight	859	47.3	242	46.8	155	43.3		
	obesity	496	27.3	156	30.2	119	33.2	0.043	0.026

The LDL-c, HDL-c and TG levels of patients with the 0-4 years duration are 3.53 ± 0.96 mmol/L, 1.25 ± 0.32 mmol/L, 2.17 ± 1.72 mmol/L. The LDL-c, HDL-c and TG levels of patients with the 5-9 years duration are 3.56 ± 0.98 mmol/L, 1.23 ± 0.31 mmol/L, 2.37 ± 1.79 mmol/L. The LDL-c, HDL-c and TG levels of patients with the ≥ 10 years duration are 3.56 ± 0.961.19 mmol/L, 1.26 ± 0.36 mmol/L, 2.38 ± 1.89 mmol/L. 53.8% of patients in 0-4 years duration is with abnormal LDL-c, 10.4% is with abnormal HDL-c, and 51.5% is with abnormal TG, while 47.2% is overweight and 27.3% is obesity; 54.6% of patients in 5-9 years duration is with abnormal LDL-c, 12.8% is with abnormal HDL-c, and 57.1% is with abnormal TG, while 46.8% is overweight and 30.2% is obesity; 56.9% of patients in 10 years or more duration is with abnormal LDL-c, 12.6% is with abnormal HDL-c, and 53.6% is with abnormal TG, while 43.3% is overweight and 33.2% is obesity. The proportion of patients with LDL-c ≥ 4.15 mmol/L is increasing with the increasing duration among the three groups while the proportion of patients with 3.46 mmol/L ≤ LDL-c < 4.15 mmol/L is decreasing. The proportion of obese people is increasing with the increasing duration among the three groups.

### Distribution of correlated factors and diseases in patients with different durations of dyslipidemia

Nineteen major factors were enrolled in the initial univariate analysis. These factors were living area, gender, educational degree, age group, occupation, current smoking status, status of breakfast, alcohol intake, status of physical activities, oil intake, degree of salt intake, low-intake of calcium, low-intake of vegetable, inefficient protein intake, degree of total cholesterol, degree of HDL-C, degree of TG, degree of LDL-C, BMI. In the same way, the correlated diseases (hypertension, diabetes, myocardial infraction, stroke, metabolic syndrome and centripetal obesity) of dyslipidemia were chosen to perform univariate analysis in order to observe the assembling mode. The significant statistic results were shown in Table 2 and Table 3.

**Table 2:** Distribution of correlated factors in patients with different durations of dyslipidemia

	code		0~4 years		5~9 years		10 years~		Statistic	P value
							
			n	%	n	%	n	%		
career	CARE1	worker	188	76.1	34	13.8	25	10.1		
	CARE2	civil servant	834	69.7	252	21.1	111	9.3		
	CARE3	resident	464	57.0	174	21.4	176	21.6		
	CARE4	farmer	157	74.0	33	15.6	22	10.4		
	CARE5	civil servant	173	78.2	24	10.9	24	10.9	77.07	<0.001
age	AGE1	18~	291	84.6	41	11.9	12	3.5		
	AGE2	40~	629	74.7	150	17.8	63	7.5		
	AGE3	50~	597	63.7	203	21.7	137	14.6		
	AGE4	60~	209	55.8	83	22.1	83	22.1		
	AGE5	70~	90	46.7	40	20.7	63	32.6	0.24	<0.001
site	SUB	Urban	1183	65.0	362	19.9	275	15.1		
	CITY	Rural	633	72.7	155	17.8	83	9.5	-4.31	<0.001
smoking group	SMK0	none	1305	69.5	342	18.2	230	12.3		
	SMK1	1~/d	134	62.6	45	21.0	35	16.4		
	SMK2	10~/d	160	66.4	47	19.5	34	14.1		
	SMK3	20~/d	159	61.4	64.	24.7	36	13.9		
	SMK4	30~/d	57	58.2	19	19.4	22	22.4	0.07	<0.001
Exercise	LACK-S	Lack	1191	65.7	357	19.7	265	14.6		
	ACT	enough	621	71.4	156	18.0	92	10.6	-3.21	0.001
brfst	BRFST1	none	116	73.0	29	18.2	14	8.8		
	BRFST2	1-3 t/w	109	73.6	25	16.9	14	9.5		
	BRFST3	4-6 t/w	129	75.0	31	18.0	12	7.0		
	BRFST4	everyday	1462	66.1	432	19.5	318	14.4	0.69	<0.001

**Table 3:** Distribution of the correlated diseases in patients with different duration of dyslipidemia

	code		0~4 years		5~9 years		10 years~		Statistic	P value
							
			n	%	n	%	n	%		
HP	HP0	normal	400	78.3	73	14.3	38	7.4		
	HP1	borderline	648	66.9	199	20.5	122	12.6		
	HP2	low-grade	492	65.3	152	20.2	109	14.5		
	HP3	middle-grade	197	60.2	66	20.2	64	19.6		
	HP4	high-grade	69	57.5	26	21.7	25	20.8	0.12	<0.001
DM	DM0	normal	1341	70.1	350	18.3	223	11.7		
	IFG	IFP	171	67.6	49	19.4	33	13.0		
	DM	DM	289	57.4	114	22.7	100	19.9	0.10	<0.001
MS	MS0	not happened	1164	70.3	294	17.8	197	11.9		
	MS1	happened	628	62.5	219	21.8	158	15.7	-4.21	<0.001
AMI	AMI0	not happened	1735	68.5	481	19.0	315	12.5		
	AMI1	happened	32	38.1	20	23.8	32	38.1	-6.57	<0.001
STROKE	STR0	not happened	1699	68.6	475	19.2	304	12.2		
	STR1	happened	75	50.0	28	18.7	47	31.3	-5.55	<0.001

### Multiple correspondence analysis on the durations of dyslipidemia and correlated factors

**Figure 1:**
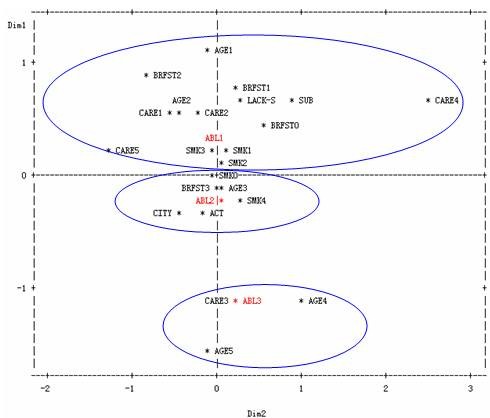
**Multiple correspondence analyses of the duration of dyslipidemia and its correlated factors. **The relevant abbreviations existed in Figure 1 are according to the codes presented in Table 2.

We excluded the insignificant factors in univariate analysis, and carried out multiple correspondence analysis on durations of dyslipidemia. Multiple correspondence analysis graph was shown in Fig 1. From the distance between codes which present the three durations of dyslipidemia and their risk factors at different level shown in Fig 1, we found that patients with duration of dyslipidemia less than 5 years were correlated with the identities of living in rural area, young to middle aged, worker, civil servant or farmer, not eating or irregular eating breakfast, less physical exercise, occasionally or often smoking; Patients with duration of 5-9 years were mainly assembled with living in urban area, 50-59 years old, eating breakfast every day, no smoking or heavy smoking, doing more physical exercises; And patients with duration of dyslipidemia ≥ 10 years concentrated mainly on retired or unemployed citizens, aged above 60 years old.

**Figure 2:**
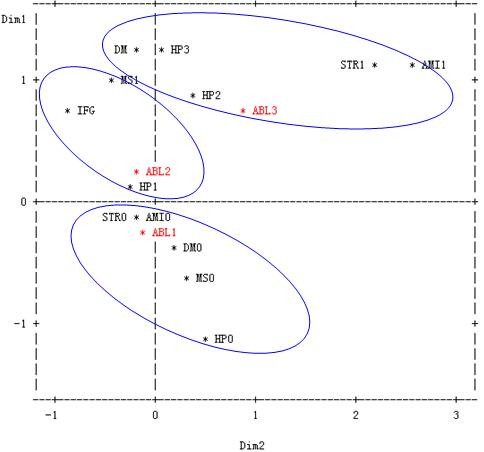
**Multiple correspondence analyses of the duration of dyslipidemia and its correlated diseases. **The relevant abbreviations existed in Figure 2 are according to the codes presented in Table 3.

We selected the significant statistic factors listed in table 2 to perform multiple correspondence analysis. Multiple correspondence analysis graph was shown in Fig 2. From the distance between the durations of dyslipidemia and their risk factors at different level according to Fig 2, we can find that patients with the duration less then 5 years was mainly assembled with normal in metabolism, blood glucose and blood pressure, without myocardial infarction stroke or centripetal obesity. Patients with the duration of dyslipidemia between 5-9 years gradually depart from normal status with critical hypertension, impaired fasting glucose(IFG), and get close to metabolic syndrome. And patients with duration more than 10 years were much closed with hypertension, diabetes mellitus(DM), myocardial infarction and stroke.

## Discussion

As an extension to principal component analysis, correspondence analysis is a kind of descriptive multi-variable analysis method, factor analysis and canonical correlation analysis. Through projection from vector point of high dimensional space to lower dimensional space, multiple correspondence analysis can be used to clearly illustrate correlations among multiple variables in one simple two dimension chart through projection from vector point of high dimensional space to lower dimensional space, therefore, can generate some benefits like that intuition, convenience, strong discriminating ability and saving calculating time, etc[15,16]. In our study the multiple variables assemble obviously.

The INTERHEART study also showed that nine traditional risk factors (smoking, hypertension, diabetes, obesity, diet, physical activity, alcohol consumption, psychosocial factors, and dyslipidemia) contributed to the high CHD burden in the South Asian population as in other countries. From the review of the report 'the nutrition and health status of Chinese' which was published by medical ministry of People's Republic of China in Oct, 2004, we knew that the prevalence of chronic disease such as hypertension, diabetes and dyslipidemia ascended rapidly and the unhealthy lifestyle was the main risk factor. Many researches had mentioned that direct or indirect smoking was one of the most important risk factors of dyslipidemia[17-19]. And the same Lack of physical activities and the habit of sedentariness were another risk factor of dyslipidemia[20]. And study suggested high prevalence of dyslipidemia in all age groups both in males and females and the prevalences were increasing with age[21]. In our study we also found that patients with shorter duration were closely related with lack of physical activities and having the habit of smoking. But the elder patients began to pay more attention to their health. And they tried to change their unhealthy lifestyle such as eating breakfast regularly, giving up smoking and doing more physical exercises, but metabolic syndrome had taken place in this period and critical hypertension and IFG happened during this period.

Tenkanen L' data showed that patients with dyslipidemia and features associated with the metabolic syndrome(BMI and TG in the highest tertiles)[22]. Hypertension, diabetes, and dyslipidemia are all factors individually associated with increased risk for mortality from cardiovascular disease and all-cause mortality[23]. Some type of diet was tested in clinical trials in Italy and shown to lower blood pressure and improve dyslipidemia[24]. Forsythe's study had suggested that a healthier diet favorably and strongly affects dyslipidemia and hypertension, even in obese patients who do not lose weight[20]. It has been indicated the combination of aerobic and resistance exercise may provide greater benefit in people with dyslipidemia and other components of the metabolic syndrome because of the combined effects of reduced adiposity, increased muscle mass, and improved myocyte function, including increased oxidative capacity[25]. According to the characteristics of patients with different duration of dyslipidemia in Beijing, We found the clues for the strategies about the prevention of dyslipidemia. At the earlier of dyslipidemia, patients were relatively young, lack of healthy sense, with the unhealthy life-style. With the development of dyslipidemia patients gradually suffered from other chronic diseases and they began to pay more attention to their health and change their unhealthy life-style. But with the effects of multiple causes myocardial infarction and stroke were inevitable. According to those characters, the strategies about the prevention of should be developed with changing young people's risk behavior. Because of the clustering of dyslipidemia, diabetes, hypertension and obesity, a comprehensive strategy should be made to improve the prevention of cardiovascular disease.

Because many different risk factors affect dyslipidemia patients with different duration, potentially complementary mechanisms of action, combination control may offer additional beneficial effects to patients with different duration of dyslipidemia. This study demonstrates that different duration of dyslipidemia with different risk factors which can effectively improve multiple intervention measures to a greater extent in patients with dyslipidemia, without significantly increasing the risk for adverse events commonly associated with unified intervention model. These findings in our paper have important public health implications for the prevention and treatments of dyslipidemia.

## Conclusions

According to our research, we should strengthen the tertiary prevention and improve the control rate of dyslipidemia in Beijing. Community physicians should take effective measures to control blood lipids for patients with dyslipidemia. We should strengthen the prevention and treatment of dyslipidemia for younger people, Particularly for the working groups and community retired residents. Health promotion programs such as tobacco control and physical exercise should be carried out for younger patients. Community hospitals should establish files with chronic diseases patients. The blood lipids, blood glucose, blood pressure should strictly controlled for the dyslipidemia patients with the duration of more than five years and then we can prevent the serious complications such as myocardial infarction and stroke.

## Competing interests

The authors declare that they have no competing interests.

## Authors' contributions

XG and PZ designed the studies, PZ participated in all data interpretation, and YL drafted the manuscript. WW, LZ, HW, WW critically reviewed the manuscript. YL carried out statical analysis. All authors have read and approved the final manuscript.
